# Exercise training restores the cardiac microRNA-1 and −214 levels regulating Ca^2+^ handling after myocardial infarction

**DOI:** 10.1186/s12872-015-0156-4

**Published:** 2015-12-09

**Authors:** Stéphano Freitas Soares Melo, Valério Garrone Barauna, Vander José Neves, Tiago Fernandes, Lucienne da Silva Lara, Diego Robles Mazzotti, Edilamar Menezes Oliveira

**Affiliations:** Laboratory of Biochemistry and Molecular Biology of the Exercise, School of Physical Education and Sport, University of Sao Paulo, Av. Professor Mello Moraes, 65- Cidade Universitária, Sao Paulo, Brazil; Laboratory of Molecular Physiology, Health Sciences Center, Federal University of Espírito Santo, Vitória, Brazil; Institute of Biomedical Sciences, Federal University of Rio de Janeiro, Rio de Janeiro, Brazil; Department of Health Informatics, Federal University of São Paulo, Sao Paulo, Brazil

**Keywords:** Exercise training, Myocardial infarction, MicroRNA, Cardiac function

## Abstract

**Background:**

Impaired cardiomyocyte contractility and calcium handling are hallmarks of left ventricular contractile dysfunction. Exercise training has been used as a remarkable strategy in the treatment of heart disease. The microRNA-1, which targets sodium/calcium exchanger 1 (NCX), and microRNA-214, which targets sarcoplasmic reticulum calcium ATPase-2a (Serca2a), are involved in cardiac function regulation. Thus, the aim of this study was to evaluate the effect of exercise training on cardiac microRNA-1 and −214 expression after myocardial infarction.

**Methods:**

Wistar rats were randomized into four groups: sedentary sham (S-SHAM), sedentary infarction (S-INF), trained sham (T-SHAM), and trained infarction (T-INF). Exercise training consisted of 60 min/days, 5 days/week for 10 weeks with 3 % of body weight as overload beginning four weeks after myocardial infarction.

**Results:**

MicroRNA-1 and −214 expressions were, respectively, decreased (52 %) and increased (54 %) in the S-INF compared to the S-SHAM, while exercise training normalized the expression of these microRNAs. The microRNA targets NCX and Serca-2a protein expression were, respectively, decreased (55 %) and increased (34 %) in the T-INF group compared to the S-INF group.

**Conclusions:**

These results suggest that exercise training restores microRNA-1 and −214 expression levels and prevents change in both NCX and Serca-2a protein and gene expressions. Altogether, our data suggest a molecular mechanism to restore ventricular function after exercise training in myocardial infarction rats.

## Background

Exercise training (ET) is a well-known therapeutic strategy used in humans and animal models to overcome the cardiovascular deleterious effects after myocardial infarction (MI) [1, 2]. In humans, ET post-MI has favorable effects on left ventricular (LV) remodeling while improving LV functional capacity, ejection fraction, and early LV diastolic filling [2, 3]. In animal models, the cardioprotective effects of ET post-MI consist of a series of beneficial effects such as reduction of total collagen content [4] and restored intracellular Ca^+2^ handling, Ca^+2^ sensitivity, and contractile function in isolated cardiomyocytes [1].

Several *in vivo* and *in vitro* studies have shown that microRNAs (miRNAs) may regulate a myriad of cellular processes, including growth, fibrosis, cell death, and neovascularization [5–10]. MiRNAs are small non-coding RNAs that regulate post-transcriptional mRNA expression mainly by binding to 3′-untranslated region (3′-UTR) of the complementary mRNA sequence, resulting in translational repression and gene silencing. Some important issues have been highlighted regarding the participation of miRNAs in gene regulation after MI [11–14]. Also, the modulation of miRNA by ET and its association to LV remodeling has recently been reviewed by us and other researchers [15–18].

In an elegant study, Van Rooij [14] showed that downregulation of miRNA-29 increased collagen expression and fibrosis in the heart after MI. Recently, our group showed that swimming training increases cardiac miRNA-29c expression and decreases collagen expression in the heart of healthy rats [18] and in the board and remote regions of the myocardium after MI [17], which is in accordance with the improved LV compliance observed after ET. In another study, Yang [19] found increased expression of miRNA-214 in cardiac tissue of patients with valvular diseases. Additionally, they observed *in vivo* that silencing miRNA-214 prevented cardiac hypertrophy and LV dysfunction in a pressure-overload mouse model of heart failure. However, the exact function of miRNA-214 in cardiac function has not yet been shown.

Several studies have shown impaired intracellular Ca^2+^ handling after MI due to altered expression and function of the NCX (sodium/calcium exchanger 1), Serca-2a (sarcoplasmic reticulum Ca^+2^ ATPase-2a), and phospholamban (PLP) in cardiomyocytes while these proteins are restored by ET [1, 20–22]. Thus, the purpose of this study was to investigate the effects of ET post-MI on the expression of cardiac miRNA-1 and −214 and their target genes NCX and Serca-2a in the remote region myocardium (RM).

## Methods

### Animal care

Male Wistar rats (body weight between 250 and 300 g and 10 weeks old) were housed in standard cages with food and water *ad libitum.* All the protocols and surgical procedures were in accordance with the guidelines of the Brazilian College for Animal Experimentation and were approved by the Ethics Committee of the School of Physical Education and Sport of the University of São Paulo (n° 2010/07).

### Experimental design and exercise training protocol

The rats were anesthetized, intubated via tracheotomy, and placed under a rodent respirator apparatus (Harvard model 680). The heart was exposed through left thoracotomy between the fifth and sixth ribs. In animals in which the MI was produced, a 9–0 Ethilon suture was placed under the left main coronary artery at a point 1–2 mm distal to the edge of the left atrium, and the artery was ligated. Sham-operated animals underwent the same procedure, except that the suture under the coronary artery was left untied. The heart was then returned to its normal position and the thorax immediately closed [23].

After 4 weeks of infarct surgery, the rats were randomly assigned to either a sedentary or training group as follows: sedentary-sham (S-SHAM; *n* = 7), sedentary infarct (S-INF; *n* = 7), trained-sham (T-SHAM; *n* = 7), and trained infarct (T-INF; *n* = 7). ET consisted of exercise sessions of 60-min duration, 5 days/week with 3 % body overload for 10 weeks. This mild-intensity long-period ET protocol has been used in our laboratory and is effective for the promotion of cardiovascular adaptations [17].

### Oxygen uptake measurements

Oxygen uptake (VO_2max_) was measured by means of expired gas analysis during the graded treadmill exercise. VO_2max_ was determined during a maximal exercise test adapted from Musch et al. [24]. The parameters were measured using the Sable Systems FC-10a Oxygen Analyzer (Sable Systems, Henderson, NV, USA). The test was carried out after a one-day recovery period from the last exercise session. The volume of the air supplied was 3.5 l/min. The gas analyzer was calibrated with a reference gas mixture before each test. The VO_2max_ test protocol involved a stepwise increase in the treadmill speed as follows: after a 15-min period of acclimation, the treadmill was started at 6 m/min, and the speed was increased by 3 m/min every 3 min until the rat was exhausted. Exhaustion was defined as spending time on the shocker plate without attempting to re-engage the treadmill within 10 s.

### Measurement of Ca^2+^-ATPase activity

The RM of the LV was excised following previous work by our group (Melo 2014); washed in a cold solution (4 °C) containing 137 mM NaCl, 2.7 mM KCl, 11.9 mM NaHCO_3_, 0.36 mM Na^+^-phosphate, 5.5 mM glucose, 1.8 mM CaCl_2_, and 0.4 mM MgCl_2_; equilibrated with a carbogen gas mixture (95 % O2/ 5 % CO2) (pH 7.4); and immediately stored at −80 °C until use. On the day of preparation, the tissue was minced and homogenized using an Ultraturrax disperser (IKA Works, Inc.) 9500 rpm for 10 s three times, with a 20 s interval between steps, in a cold solution containing 0.2 mM phenylmethylsulfonyl fluoride, 2 mM dithiothreitol, 0.4 μg/mL aprotinin, 0.2 mM EDTA, and 250 mM sucrose in a 5 mM Tris–HCl (pH 7.4). The crude preparation was ultracentrifuged at 110,000 g for 35 min. The pellet was re-suspended in the same buffer without EDTA and dithiothreitol and stored at −80 °C, and protein concentration was determined [1].

Homogenate fractions obtained from cardiac tissue (0.2 mg/mL, final protein concentration) were incubated for 1 h in a medium (0.5 ml, 37 °C) containing 50 mM MOPs-Tris (pH 7.4), 5 mM NaN_3_, 0.2 mM EGTA, 1 mM [γ-^32^P] ATP (disodium salt; specific activity ~1.5 × 1010 Bq/mmol), 4 mM MgCl_2_, and 100 mM KCl, in the presence or absence of 3 μM thapsigargin. The total CaCl_2_ needed for the free Ca^2+^ concentration of 50 μM was calculated according to [25]. Thapsigargin-sensitive Ca^2+^- ATPase activity was determined by the difference between the Ca^2+^-ATPase activity measured in the absence (total Ca^2+^- ATPase) and presence of thapsigargin. The thapsigargin-resistent Ca^2+^- ATPase activity was then related to the plasma membrane Ca^2+^-ATPase (PMCA). Reactions were stopped by adding 1 ml cold 26 % (w/v) charcoal in 0.1 N HCl. The tubes were centrifuged at 1,500 × g for 15 min at 4 °C, and 0.25 ml of supernatant containing the released ^32^Pi was counted by liquid scintillation.

### mRNA and miRNA quantification using real-time PCR

Frozen RM of the LV samples were homogenized in trizol, and ribonucleic acid (RNA) was isolated according to the manufacturer’s instructions (Invitrogen Life Technologies, Strathclyde, UK). cDNA for miRNA-1, miRNA-214, and U6 analysis were synthesized according to the TaqMan MicroRNA Assay protocol for miRNA-1 (ID 2222), −214 (ID2223), and U6 (#4373381).

The mRNA expression of alfa actin, ANF, Serca-2a, and NCX was assessed by oligonucleotides primers as follows: α-Actin, 5′-GCTCTTTCCAGCCTTCCTTT-3′ and 5′-ACGTTGTTGGGTACAGGT-3′; ANF, 5′-CTTCGGGGGTAGGATTGAC-3′ and 5′-CTTGGGAATCTTTTGCGATC-3′; Serca-2a, 5′- CTCCTTGCCCGTGATTCTCA-3′ and 5′-CCAGTATTGCAGGTTCCAGGTA-3′ and NCX, 5′-GGGATTTCAGCTCTGCTACTCA-3′ and 5′-GGCTTGCCCATCTCTGCTAT-3′. The expression of cyclophilin A 5′-TGGCAAGCATGTTGGGTCTTTGGGAG-3′ and 5′-GGTGATCTTCTTGCTGGTCTGCCATTC-3′ was measured as an internal control. Quantification of the target genes expression was performed with a SYBRgreen PCR Master Mix (Applied Biosystem, PE, Foster City, CA, USA). The relative expression of the mRNA and miRNA were performed by real-time PCR in the ABI PRISM 7700 Sequence Detection System (Applied Biosystem).

### Western blotting

The RM of the LV (100 mg) was thawed, minced into small pieces, and homogenized in cell lysis buffer containing 100 mM Tris–HCl, 50 mM NaCl, 1 % Triton X-100, and a protease and phosphatase inhibitor cocktail (1:100; Sigma-Aldrich, MO, USA). Insoluble LV tissues were removed by centrifugation at 3,000 × g, 4 ° C, 10 min. Samples were loaded and subjected to SDS-PAGE in polyacrylamide gels (6–15 %) depending on the protein molecular weight. After electrophoresis, proteins were electro-transferred to the nitrocellulose membrane (BioRad Biosciences, NJ, USA). Equal loading of samples (30 μg) and even transfer efficiency were monitored with the use of 0.5 % Ponceau S staining of the blotting membrane. The blot membrane was then incubated in a blocking buffer for 2 h at room temperature and then incubated overnight at 4 °C with Serca-2a (catalog No. #4388) monoclonal antibody (Cell Signaling Tech., MA, USA) or total PLP (catalog No. ab86930) polyclonal antibody or corresponding phosphorylated PLP (Ser^16^) (catalog No. ab15000) polyclonal antibody or NCX (catalog No. ab2869) monoclonal antibody or GAPDH (catalog No.ab9484) monoclonal antibody (Abcam, Cambridge, UK). The bands were visualized by using a chemiluminescent detection kit (Amersham, ECL™ Western Blotting Detection). After measuring the intensity of the bands (Molecular imager, ChemiDoc XRS, Bio-Rad), the bands were analyzed using the program Scion Image software.

### Statistical analysis

Statistical analysis was performed using randomized two-way analysis of variance. The Bonferroni post hoc test was used for individual comparisons between means when a significant change was observed (Sigma software Stat). Correlation between miRNA 214 and SERCA-2 protein expression was performed by Pearson correlation coefficient. For all experiments, *p* < 0.05 was assumed for a significant difference. All results are presented as mean ± standard deviation.

## Results

Ecocardiography data showed that ET restored cardiac function in MI rats [17]. After ET protocol, the E/A ratio, the marker for diastolic function, did not differ between the S-SHAM (1.40 ± 0.1) and T-SHAM groups (1.55 ± 0.4). However, in the T-INF (1.74 ± 0.1) group we found a decrease of 17 % in the diastolic function represented by E/A ratio compared with the S-INF group (2.05 ± 0.1) [17].

**Fig. 1:**
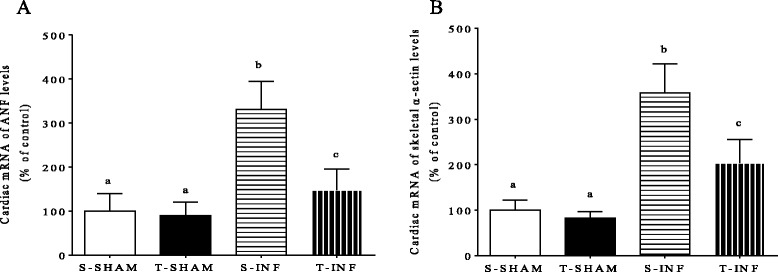
Expression of cardiac ANF and skeletal α-actin by real-time PCR, in remote myocardium of the infarcted. Percentage related to SED SHAM group, *n* = 7 each group. All results are presented as mean ± standard deviation. Different letters indicate statistically different groups (*P* < 0.05)

Analysis of the molecular marker of cardiac hypertrophy by qRT-PCR showed that MI increased 2.3-fold ANF expression and 2.5-fold skeletal α-actin expression in the S-INF compared with S-SHAM. In contrast, ANF and skeletal α-actin were decreased in the T-INF group compared with S-INF (Fig. 1a and b). These data are in agreement with previous data from our groups (REFs) and others (REFs) showing that ET induces physiological cardiac hypertrophy.

**Fig. 2:**
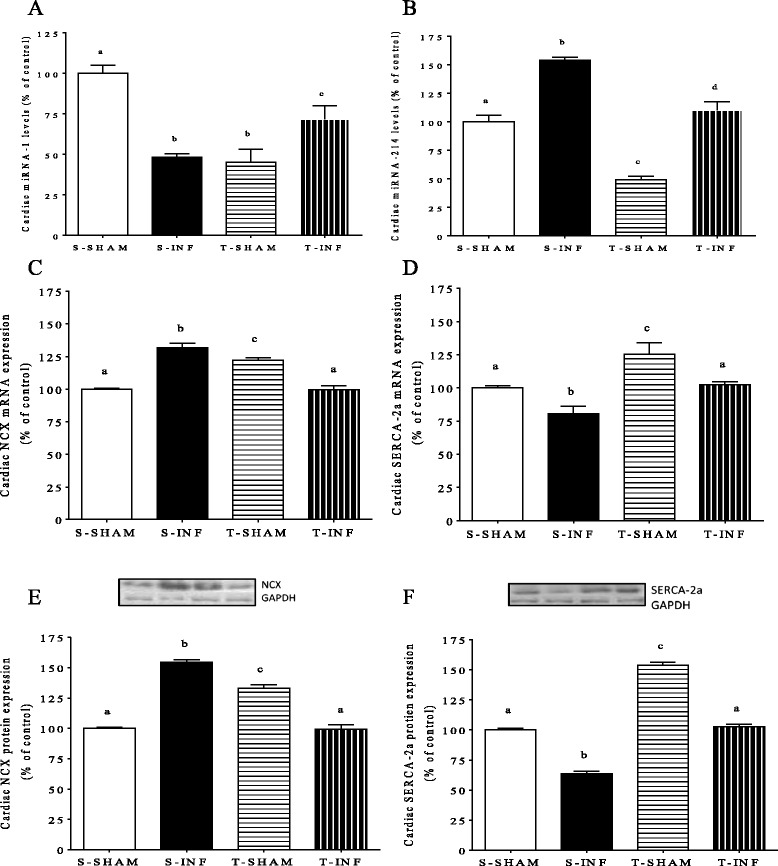
Expression of cardiacmRNA of the Serca-2a (**a**), mRNA of the NCX (**b**), miRNA-1 (**c**) and −214 (**d**) by real-time PCR reaction and expression of NCX (**e**) and Serca-2a protein expression (**f**) by western blot (blot above showed), in remote myocardium of the infarcted. Percentage related to SED SHAM group, *n* = 7 each group. All results are presented as mean ± standard deviation. Different letters indicate statistically different groups (*P* < 0.05)

Next, we looked to the expression of cardiac miRNA-1 and −214 expressions. It can be observed in Fig. 2a and b that miRNA-1 decreased 52 % and miRNA-214 increased 54 % after MI. ET protocol after MI restored both miRNA-1 (23 %) and miRNA-214 expression to basal levels (Fig. 2a and b).

**Fig. 3:**
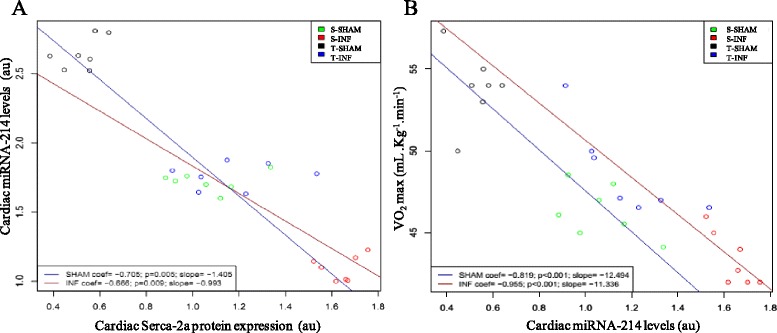
Correlation between (**a**) miRNA-214 levels and Serca-2a protein expressionby western blot and (**b**) VO_2_ max and miRNA-214 levels. Blue lines show linear regression applied for SHAM rats and red lines for INF rats, *n* = 7 each group

NCX and SERCA-2a are two important molecules involved in Ca2+ handling and targets to miRNA-1 and −214. Figure 2c and d shows mRNA expression, while 2E and F shows the protein expression. MI increased NCX gene (31.8 %) and protein (54.4 %) expressions, while it decreased Serca-2a gene (20.4 %) and protein (36.4 %) levels. Interestingly, after ET, both NCX gene and protein expression were increased (32.6 % and 55.0 %, respectively), while both Serca-2a gene and protein expression were decreased (20.9 % and 34.1 %, respectively) in the T-INF group compared with S-INF (Fig. 2c, d, e, and f). Strong negative correlation was observed between Serca-2a and miRNA-214 (Fig. 3a) and between VO_2max_ and miRNA-214 (Fig. 3b).

**Fig. 4:**
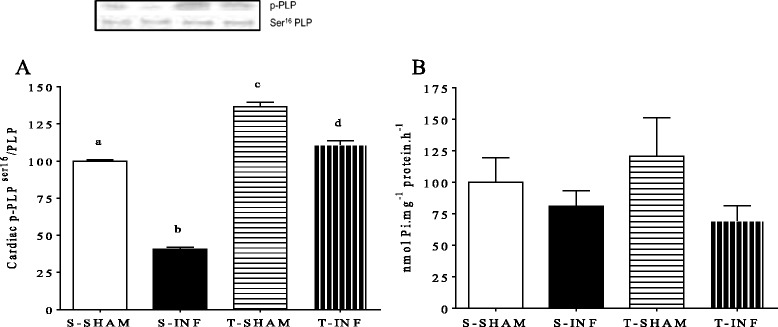
Expression of cardiac phospholamban protein expression (**a**) by western blot (blot above showed) and Serca-2a activity (**b**) by measurement of Ca^2+^-ATPase activity in remote myocardium of the infarcted. Percentage related to SED SHAM group, *n* = 7 each group. All results are presented as mean ± standard deviation. Different letters indicate statistically different groups (*P* < 0.05)

Since Serca-2a can also be post-transcriptionally modulated by PLP, we also looked to its phosphorylation status. p-PLP decreased 36.2 % in the S-INF group compared with the S-SHAM group (Fig. 4a). However, ET increased p-PLP in both the T-SHAM and T-INF groups (Fig. 4a). Unexpectedly, we did not observe any change in Ca^+2^ ATPase, although there was a tendency to decrease in the S-INF group and increase in the T-SHAM group (Fig. 4b).

## Discussion

In this study, we have demonstrated a possible molecular mechanism to explain the improved ventricular function after MI by ET. We showed for the first time a role for miRNAs on protein expression related to Ca^2+^ handling in cardiomyocytes after ET. MI induced a decrease in miRNA-1 expression and an increase in miRNA-214 expression. However, ET after MI partially restored miRNA-1 and returned miRNA-214 expression to basal levels. Among the proteins related to Ca2+ handling, NCX is a validated target of miRNA-1, while Serca-2a is a predicted target of miRNA-214. ET changed both gene and protein levels to basal levels after MI, showing that the mechanism is transcriptionally regulated. These results show that ET at least partially restored cardiac target genes related to Ca^2+^ handling. These molecular adaptations may be associated with improved diastolic function as previously published by our group in an MI model [17].

The most interesting association observed in our study was with miRNA-214 and Serca-2a. Pearson correlation showed a strong correlation between Serca-2a and miRNA-214 in both the SHAM and INF groups. Through *in silico* analysis of predicted mRNA targets for miRNA, we found a conserved region on Serca-2a mRNA to miRNA-214 binding. Serca-2a represents 90 % of the membrane proteins in the sarcoplasmic reticulum of cardiac muscle [26], is responsible for up to 92 % of Ca^2+^ reuptake in rats [21, 27], and its downregulation is used as a hallmark of cardiac dysfunction [28, 29]. Other miRNAs have already been described to regulate Serca-2a expression. Recently, Gurha [30] showed that genetic ablation of miRNA-22 indirectly modulates Serca-2a expression, and Wahlquist [31] showed that miRNA-25 also indirectly regulates Serca-2a expression and contributes to impaired cardiac function during heart failure. In our study, the recovered expression of Serca-2a by miRNA-214 may represent one factor directly responsible for the improved cardiac function after ET.

Although ET has emerged as an interesting interface to reparative function of the Ca^+2^ ATPase post-MI [1, 26, 27], Bupha-Intr [32] demonstrated that an exercise program did not induce any effect on Serca-2a activity in healthy cardiomyocytes, which is in agreement with our data. However, differently from other authors [1, 33], we have not observed any change of Ca^+2^ ATPase activity in trained rats after MI. In this sense, we may speculate that total protein expression of Serca-2a could also be crucial to provide better Ca^2+^ uptake and improve cardiac contractility after ET. In addition to miRNAs regulation, there are other important modulators of Serca-2a, such as the PLP. Dephosphorylated PLP suppresses Serca-2a pump activity, whereas phosphorylation of PLP leads to Serca-2a deinhibition and increased activity [20, 22]. Consistently with this mechanism, our data show decreased PLP phosphorylation in the S-INF group and increased PLP phosphorylation after ET in both the SHAM and INF groups.

MiRNA-1 is one of the most abundant miRNAs in the heart and regulates several gene expressions [34]. A study by Kumarswamy [35] validated NCX mRNA as a target to miRNA-1. Using gene therapy to restore miRNA-1 levels in a heart failure model, the authors showed that NCX expression was recovered to basal levels, while cardiac function was improved to a healthy condition. Here, we also showed that MI decreased miRNA-1 expression, while ET prevented its decrease at least partially, suggesting its close association to cardiac NCX protein expression that was also normalized in the T-INF group. However, it was unexpected the decrease of miRNA-1 in the T-SHAM group. Therefore, more specific studies should be done in order to understand the complexity of this regulation in both physiological and pathological situations.

miR-214 is upregulated during ischemic injury and heart failure [19], but its role in these processes is unknown. Since the NCX gene was also validated as a target to miRNA-214, increased miRNA-214 has been suggested as a mechanism to downregulate the NCX in a pathological condition. However, we observed increased the NCX and it was not association with miRNA-214 in the S-INF group. An explanation for increased NCX in the S-INF is that in some pathological situations the NCX can work in reverse mode to pump Ca^2+^ back into the cell, induce Ca^2+^-induced Ca^2+^ release from the sarcoplasmic reticulum, and lead to additional Ca^2+^ overload and cardiac dysfunction [36]. On the other hand, in the T-INF group ET prevents the increase of the miRNA-214, which was associated with the expression of NCX in S-SHAM group, a known mechanism induced by ET to prevent Ca^2+^ overload [1].

## Conclusion

In conclusion, we have demonstrated that MI is associated with altered expression of cardiac miRNA-1 and −214. Furthermore, moderate ET restored miRNA-1 and −214 expressions in the RM post-MI. These responses may be associated with the normalization of Ca^2+^ handling and LV compliance in infarcted hearts after an ET program. Therefore, it would be very interesting to confirm if these miRNAs also have a positive impact on cardiac recovery of Ca^2+^ handling in patients post-MI.
